# An affordable implantable vagus nerve stimulator system for use in animal research

**DOI:** 10.1098/rsta.2021.0010

**Published:** 2022-07-25

**Authors:** Ángel Canal-Alonso, Roberto Casado-Vara, Orlando Castellano, Jorge Herrera-Santos, Jaime Gonçalves, Sergio Màrquez-Sànchez, Jesús María Gonçalves, Juan Manuel Corchado

**Affiliations:** ^1^ Bioinformatics, Intelligent Systems and Educational Technology (BISITE) Research Group, Salamanca, Spain; ^2^ Institute for Biomedical Research of Salamanca (IBSAL), Salamanca, Spain; ^3^ Department of Cell Biology and Pathology, Faculty of Medicine, University of Salamanca, Salamanca, Spain; ^4^ Institute of Neuroscience of Castilla y León (INCYL), University of Salamanca, Salamanca, Spain; ^5^ Department of Surgery, Faculty of Medicine, University of Salamanca, Salamanca, Spain

**Keywords:** epilepsy, implantable devices, neuromodulation

## Abstract

In this research, a vagus nerve stimulator has been developed and miniaturized for use in epilepsy research. The board contains all the components necessary for its operation during the standard duration of the experiments, being possible to control it once implanted and even being able to reuse it. The VNS system has been designed for rodents since the VNS devices available for human are not only too large for laboratory animals, but also too expensive. With this solution the expenditure on materials made by laboratories is greatly reduced and bioethical considerations were kept in mind. The system was validated in hamsters.

This article is part of the theme issue ‘Advanced neurotechnologies: translating innovation for health and well-being’.

## 1. Introduction

Epilepsy is currently one of the most common and severe brain conditions, affecting at least 1% of the population, which means that over 70 million people worldwide suffer from it [1]. This condition expresses itself in several different forms and is characterized by the recurrence of unprovoked seizures.

The definition of epileptic seizures includes the transitory appearance of more or less severe signs and symptoms caused by excessive or simultaneous abnormal neuronal activity in the brain. Epilepsy is a debilitating disease with a significant negative impact on the patient’s quality of life. Therefore, its management must consider the psychosocial dimension due to the neurobiological, cognitive, psychological and social consequences that derive from epileptic seizures [2].

Fortunately, most patients can control their symptomatology with antiepileptic drugs; however, approximately 30% of patients do not respond favourably to drug treatment. The diagnosis of these patients falls into the category of refractory epilepsy, drug-resistant epilepsy or intractable epilepsy. Refractory epilepsies present complex clinical management and are frequently associated with cognitive damage, behavioural disorders and more significant impairment of the quality of life of the patient and their family [3].

There are various alternative treatments—different from antiepileptic drugs—for patients who have refractory epilepsy. Some of these alternatives are surgical treatments. However, there are patients not suitable for surgical interventions or adverse and side effects are unacceptable. In these cases, other approaches can be followed, such as treatments based on the use of magnetic or electrical stimuli, which have varied features and offer several different possibilities [4–6].

### (a) Brain stimulation in epilepsy

The stimulation of areas of the nervous system is commonly known as neuromodulation or neurostimulation. Additionally, this type of brain stimulation comprises three general branches: deep brain stimulation, cortical stimulation and extracranial stimulation. The number, classification and characteristics of these methods considerably differ, depending on the criterion of the authors. The most widely used ones, and those where clinical trials have been performed, or are on the way, are briefly discussed.

#### (i) Deep brain stimulation

Stimulation of deep brain areas has shown to be feasible in humans affected by different disorders. Consequently, there has been an increase in the development of technologies and procedures addressed to use deep brain stimulation (DBS) in a high number of neurological, psychiatric and other diseases. The general DBS procedure involves the placing or inserting of one or more electrodes in the selected target. These targets include the cerebellum, thalamic and basal ganglia nuclei, substantia nigra and piriform cortex since these subcortical structures, in addition to the cortex, play a crucial role in the origin, propagation and management of seizures [7,8].

The anterior nucleus of the thalamus has been the region with more promising results regarding the DBS treatment of epilepsy, which is why the FDA has approved its use [5]. Nevertheless, there is a need for larger and well-designed studies to help optimize the efficacy and safety of invasive intracranial neurostimulation treatments.

#### (ii) Cortical electrical stimulation

Another method used to treat epileptic seizures is cortical electrical stimulation. This procedure is commonly, but not exclusively, performed by the implantation of a subdural device. Several results show that electric pulses can reduce seizure degree or even suppress seizure activity when short-term continuous electrical stimulation in the cortex is applied. This approach, using open-loop via a cyclic or continuous manner, has also shown encouraging results for refractory epilepsy [9].

The cortical electrical stimulation procedure can be performed either in an open-loop, or in a closed-loop method, which is the most recommended in recent times. Open-loop stimulation reduced seizure frequency in the majority of patients (over 90%) [10]. At the same time, closed-loop stimulation reduces seizure frequency in a high average of 60–65% [10]. Although the procedures have only been reported in a few dozens of patients, evidence suggests that both approaches are viable choices to control refractory focal seizures.

#### (iii) Responsive neurostimulation

A more complicated but also effective and reliable system is responsive neurostimulation (RNS). This method is composed of a programmable and responsive device that consists of leads, a pulse generator and an external controller. Its functioning is based on the delivery of brief pulses of closed-loop electrical stimulation, to help normalize specific patterns of epileptogenic activity every time they are detected by an algorithm. RNS is an effective and safe adjunctive therapy that, in addition to seizure frequency reduction, may have other applications such as drug-response evaluation and long-term electrocorticography recording [11,12].

More than 2000 patients have likely been treated with RNS since the FDA approved its usage almost 10 years ago [13]. However, regardless of its use, RNS still presents diverse challenges for clinicians.

#### (iv) Transcranial magnetic stimulation

Although it seems inconsistent, some attempts have been performed through the stimulation at seizure focus, with the aim of hampering the progression of epileptic seizures. Transcranial magnetic stimulation (TMS) is a non-invasive tool for stimulating neural tissue and can be applied in different forms, both for diagnosis and treatment. When TMS is used as therapy, trains of stimuli (repetitive TMS) can modify the cerebral cortex function, both at the stimulated site and quite far from it, involving areas along with functional connections [14]. The advantage of this approach is its invasiveness. Still, this perk is usually counterpoised with the effectiveness of the procedure, which is why it is not widely used in epilepsy.

#### (v) Transcranial direct current stimulation

In the last year, Transcranial direct current stimulation (tDCS) has emerged as a promising therapeutic tool in diverse fields (addictions, rehabilitation, psychiatry, learning, memory, etc.), deemed a safe and probably effective technique for seizure control in patients with drug-resistant focal epilepsy. In this case, the stimulation is produced by placing the active electrode on the scalp over the pathologically affected region (for example, hippocampus) and the reference electrode, over the contralateral region (supraorbital region) [15]. However, published trials are heterogeneous regarding samples and methodology. Therefore, there is a need for more extensive sham-controlled randomized trials, preferably with mechanistic informed stimulation protocols, to further advance tDCS therapy in the management of epilepsy [16].

#### (vi) Vagus nerve stimulation

Equally or more important is vagus nerve stimulation (VNS). This method was introduced in humans in 1988 to manage difficult-to-treat epilepsy [17], after which it has been approved for use in many countries worldwide, benefitting more than 100 000 patients. The VNS therapy system is comprised of an electrode connected with a programmable and remote-controlled pulse generator located at the subcutaneous space in the chest wall.

Additionally, VNS has also been proposed as a therapeutic alternative in more than 20 conditions in more recent times. In some of these cases, practical applications have been made. In others, its potential use ranges from treating affections as dissimilar as inflammatory bowel disease, rheumatoid arthritis, eating or mood disorders to Alzheimer’s disease, migraines or Parkinson’s disease, to name just a few. The methods used to induce VNS can be invasive (surgical implantation of the stimulation system) and non-invasive (transcutaneous stimulation).

Undoubtedly, of all the procedures available to treat refractory epilepsy, VNS is the most widely used for the multiple benefits it offers and for the few adverse effects it has [18]. Although the mechanisms responsible for the amelioration that its use induces are not entirely clear, it is of great interest to continue developing research that allows knowing in depth all the elements that participate in such improvement. Among the various theoretical and practical postulates that support the use of VNS as a tool to reduce the number or intensity of seizures, it might be pointed out that, on the one hand, the stimulus would generate a modification in neuronal polarization, decreasing cortical excitability [19]. On the other hand, there would be an increase in the production of inhibitory neurotransmitters and a decrease in excitatory neurotransmitters. In addition, there is an increase in blood flow to the thalamus bilaterally. Additionally, an immunomodulatory and neuroprotective effect of VNS was recently demonstrated since it produces an increase in the levels of neuroprotective tryptophan metabolites and a decrease in neurotoxic metabolites [20,21].

In drug-resistant epileptic patients, electrical stimulation represents a good, safe and efficient alternative treatment. In addition, some of the varied technical advantages of VNS include reversibility and adjustability. However, contrary to resective procedures, the use of VNS remains palliative. As we pointed out before, VNS is licensed in many countries as an adjunctive therapy. However, most of the other stimulation procedures must be considered experimental, even although several controlled studies are currently under investigation.

### (b) Experimental models in epilepsy

The use of experimental models allows us to carry out research on human disorders, which is crucial for the advancement of biomedicine. Currently, there are various experimental models of epilepsy; some require the use of drugs, others are based on electrical stimulation and others are from genetic origin. Each one has its advantages and disadvantages, and its use depends on the objectives of the studies to be performed. Having good experimental models that are also reliable and cost-effective electrical stimulation systems is an essential requirement for in-depth knowledge on the matter and for advancing the development of new therapeutic procedures.

### (c) Commercial stimulation devices

The most widely used stimulation method in humans is VNS. Therefore, we will cover the latest advances in VNS devices and their drawbacks when used in experimental models.

Most VNS devices that are implanted surgically can work in an open-loop or closed-loop fashion. Open-loop stimulation refers to devices with preprogrammed parameters, while closed-loop stimulation devices change their parameters dynamically to adapt to the situation of the patient. This parameter adaptation is based in the monitorization of some physiological parameters such as cardiac activity or body position.

Although commercial stimulation devices are effective and safe, their average cost is too high to be used as a research solution in animal experimentation [22]. Also, most of them have a large number of features that complicate the design but are not necessary for experimentation. This excess of features usually implies a large size, not suitable for implantation in smaller organisms such as experimental mice or hamsters.

## 2 Methods

**Figure 1.**
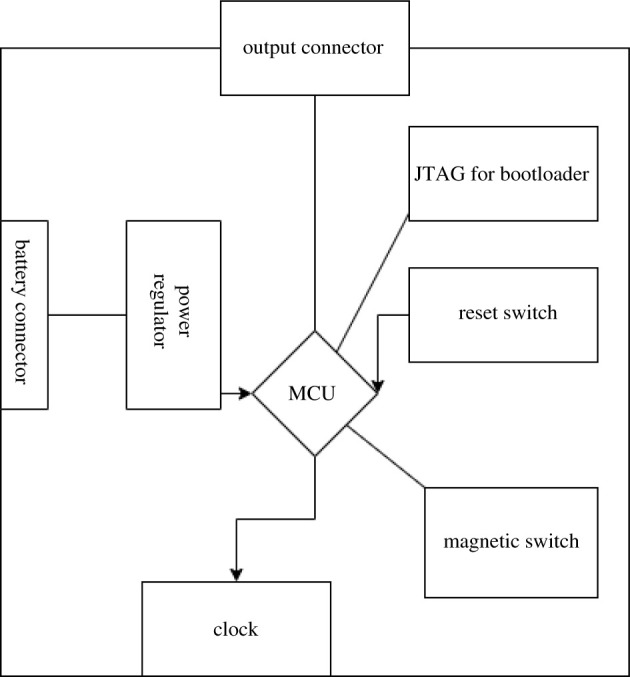
Concept diagram with the intended modules to include in the device as stated in the proposed objectives.

This section will explain the testing and the basic development techniques that have been used in the research. The initial diagram of the concept is shown in figure 1, used as a starting point for the work.

### (a) Consumption tests

A DC power supply and a precise multimeter were used to check the consumption of the PCB. It has been verified that when the device is asleep it consumes 10 μA and when operational it consumes 10 mA, being the average consumption of 1 mA. At first, it was thought to use a rechargeable LiPo battery, but for capacities above 500 mAh the size was too big so was discarded to keep the device small. It was therefore decided to use a 530 mAh 3.3 V button cell battery, as it provides the necessary operating voltage for the PCB and provides sufficient power.

After working on optimizing the microcontroller code, an average power consumption of 18.45 μA has finally been achieved. That implies that even the smallest button cell batteries on the market can be used. In our case, it was decided to choose a commercial CR2012 button cell battery of 55 mAh, which provides an autonomy of 372.13 days.

### (b) In silico testing

To check that it works correctly, a logic analyser is coupled to the output pin of the device to check whether the board is generating the signal or not. A precise multimeter connected between the battery and the device is used to check the input intensity current flow when the device is asleep or working normally.

### (c) *In vivo* testing

VNS surgery protocol used in the clinic was adapted to the experimental model of epilepsy genetic audiogenic seizure hamster from the University of Salamanca (GASH/Sal) [23]. Traditional VNS in patients requires a surgical intervention, in which a bipolar electrode is placed around the left vagus nerve. This electrode is connected to a pulse generator that is implanted subcutaneously in the chest wall. This surgery is performed by a neurosurgeon and takes approximately 2 hours under general anaesthesia [24].

VNS surgery shows low risk, although haemorrhages or infections can occur. Thus, most of the side effects of this treatment are mainly associated with the therapy, not due to surgery. Symptoms such as hoarseness, coughing or sore throat are often mild, transient and present only in the ON phase of stimulation. Therefore, these symptoms can usually be controlled by changing stimulation parameters, and their presence decreases over time [25].

Although it is a simple procedure, some technical considerations need to be made regarding electrode placement. Firstly, the cathode is placed cranially, whereas the anode is oriented caudally. This orientation is thought to enhance stimulation of the afferent fibres that project to the brain and block action potentials that travel through the efferent pathway. However, it has been demonstrated that not all these action potentials are blocked. Secondly, VNS is applied to the left vagus nerve in order to avoid adverse cardiovascular side effects since the right vagus nerve has more projections to the cardiac atria. However, stimulation of the right vagus nerve has shown to have an antiepileptogenic effect as well [25].

To mimic clinical settings in our experimental model, surgery was performed under aseptic conditions using inhalation anaesthesia with 4% sevoflurane, 1 L/min O_2_ for sedation and 3% sevoflurane 1 L/min O_2_ for maintenance (Forane, Abbot Laboratories). Physiological serum was applied periodically to avoid ocular desiccation. Additionally, the use of a thermal blanket prevented heat loss.

**Figure 2.**
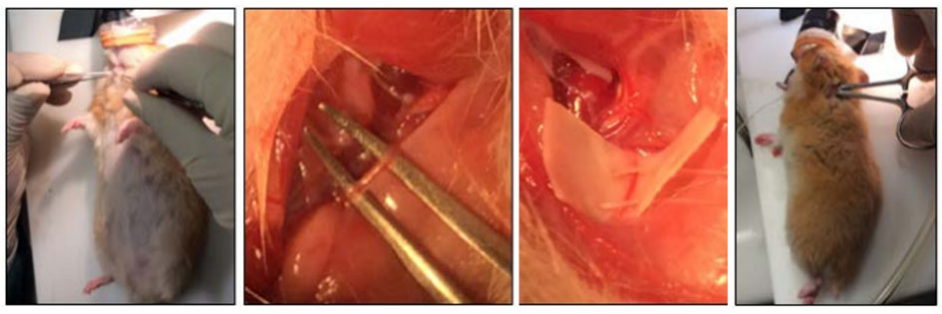
Images of the surgery to implant the electrodes and the device. (Online version in colour.)

For the surgery in animals, first of all, a 1 cm long incision was made in the left anterolateral cervical area to expose the left vagus nerve at the intersection of sternocleidomastoid and sternohyoid muscles and adjacent to the carotid artery. Under the surgical microscope, the vagus nerve was bluntly dissected using gentle opening–closing movements to avoid puncturing blood vessels. Moreover, a sterile micropatch was placed around the cervical vagus for nerve isolation to prevent damaging it and other neighbouring structures during its direct manipulation. This procedure can be seen in figure 2.

Secondly, the animal was placed in prone position, and a small incision was made in the posterior part of the neck in order to implant the neurostimulation device. This procedure enabled the bipolar electrode to be tunnelled to the left anterolateral cervical surgical area and to be placed around the nerve. Next, the electrode was fixed to the nerve by knotting a non-absorbable suture around its case. Finally, both incisions were closed after checking the cuff was encircling the nerve correctly without twisting or pulling and after creating a subcutaneous pocket for the pulse generator in the animal’s dorsum.

After surgery, the animals had a recovery period with water and food ad libitum in individual cages and received buprenorphine subcutaneously (0,5 mg/kg) as post-operative analgesia during the first 3 days. Given the size of the hamster vagus nerve, a bipolar electrode was designed for this purpose (MicroProbes For Life Sciences, MD, USA). It is essential to use an appropriately sized electrode to prevent nerve damage and current leakage, which might happen if the electrode placed is too big or too small. Finally, the nerve cuffs consisted of two rings with an inner diameter of 500 μm each, with an individual contact made of 50 um platinum/iridium wire. Leads were 70 mm long with insulation.

## 3. Results

### (a) Final proposed device

**Figure 3.**
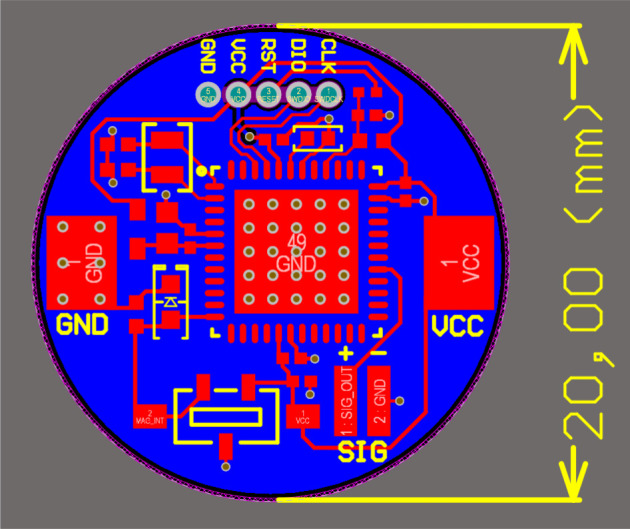
Final layout of the prototype. (Online version in colour.)

**Figure 4.**
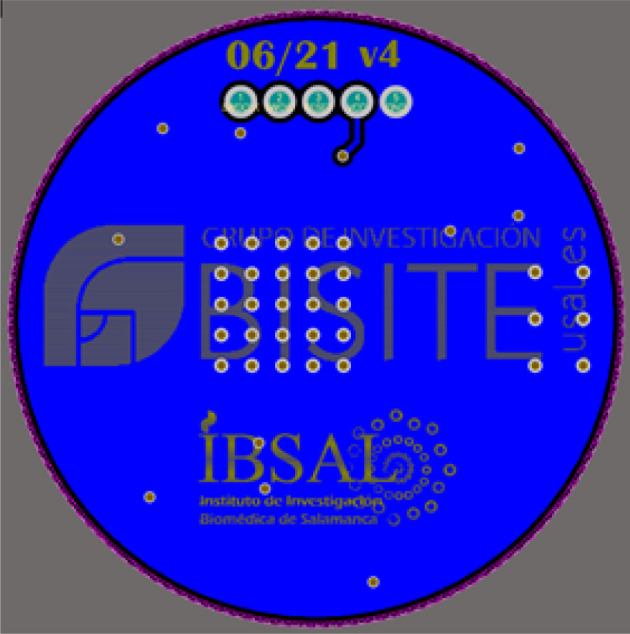
Bottom view of the final prototype. (Online version in colour.)

After careful consideration and the evaluation of the different iterations of the designed device the final layout for the prototype was selected to be as in figures 3 and 4.

The main changes introduced after the different versions were the shape (from rectangular to round), the size (reducing it considerably) and the batteries (improving the duration and size by optimizing the code and, therefore, the time that the device is kept awake, which means lower consumption). Also, a three-dimensional printed coating has been prepared to enhance the properties of the device.

### (b) Board schematics

**Figure 5.**
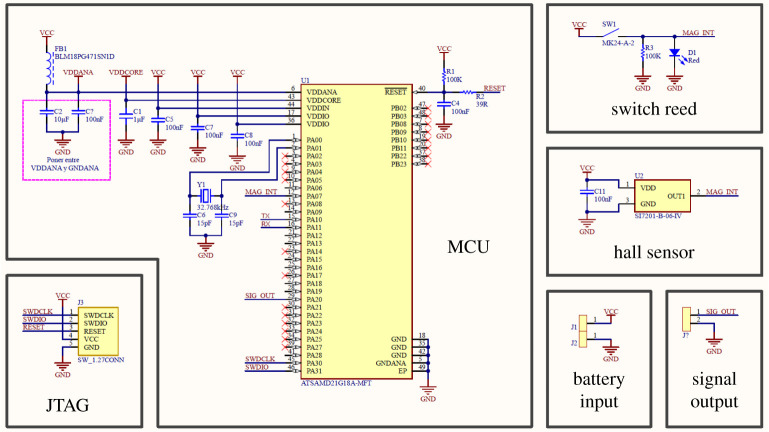
Schematics of the final version of the device. (Online version in colour.)

The schematics of the PCB can be seen in figure 5.

The first part of the circuit is the microcontroller unit (MCU). This board uses an ATSAMD21G18A as the main microprocessor. This microprocessor has been chosen because it allows more flexibility when modifying the timer registers of the MCU and to get a wide range of possible frequencies to the pulse width modulation (PWM) outputs; also, due to the low consumption that it has when it works in sleep mode, it also allows the direct communication between it and a computer by serial port for its programming or the sending and receiving of information without the need of a chip that fulfils the function of the USB interface. This part includes the capacitors and filter coils as well as the crystal with a frequency of 32.7 KHz for running a real time clock (RTC), needed to measure the time correctly.

In the JTAG, there is a five pin connector with a separation of 1.27 mm. It will be used to upload the code to the microcontroller.

Two connectors are included, one that will be for the battery to feed the PCB and another that will be for the probe that carries the signal output.

A magnetic switch has been added to keep the microcontroller in stand-by mode before being inserted into the animal. Once the surgery is done, the doctor approaches a magnet so the switch activates and the device wakes up due to a change of logic level at the magnetic switch input.The sensing range of the Hall sensor is ±2.0 mT for trip and ±0.6 mT for release.

### (c) Board layout

The distribution of the components on the initial prototype PCB is mainly marked by the size of the PCB and the need to have the connectors in accessible areas.

Although in an initial design both sides of the PCB were used for the capacitors and coils associated with the MCU, in the final version all the components are on the top side of the PCB so the button cell battery can be located in the bottom side.

A circular design is used in the prototype to reduce the edges and facilitate implantation and to ensure that the size of a non-rechargeable button cell battery (20 mm in diameter) can be adapted. The distribution of the components is conditioned firstly by the space, and secondly by the whole circuit to which each component belongs. The power connectors consist of two copper areas, located at the edges of the PCB, where two nickel strips that connect the two poles of the battery (GND and 3 V) are connected.

### (d) Code

The way the code will be explained is the following, firstly the bootloader used as well as the libraries used and the global variables, then the set-up, the loop and the two functions associated with the interrupts will be explained.

#### (i) Set-up

The basic functions of the microcontroller are configured.

— GPIOs: Unused input or output pins are disabled. The pin connected to the magnetic switch is configured as an external interrupt input and the pin connected to the probe is configured as an output.
— PWM: The PWM function, used to induce the electromagnetic pulse in the vagus nerve of the mouse, is set to a frequency of 30 Hz, with a signal high time of 250 ms.
— RTC: The RTC is set to produce two interruptions: one every 30 s and one every 4.30 min. The 4.30 min interrupt is used to wake up the microcontroller and enable the PWM pulse generation and start the 30 s counter. The 30 s interrupt is used to disable the PWM signal and induce the microcontroller in a low power state (stand-by).

Once configured, the device is kept in a low-power state, from which it will be awakened by an external interrupt (magnetic switch).

#### (ii) Loop

Like all code programmed for microcontrollers, it consists of an infinite loop, which will run until the device runs out of battery power.

This loop starts once the external interrupt has been triggered, which means that the surgery has been completed and the doctor has brought a magnet close to the device. The PWM signal is then enabled, the 30 s counter is enabled and the device enters the low-power stand-by state. Thanks to the composition of the SAMD21 microcontroller, the microcontroller will be able to continue generating the signal even in a low power state. Once the 30 s have elapsed, the PWM signal generation function is disabled, the 4.30 min counter is started and the microcontroller is induced into a low power state. The loop will repeat after this time.

### (e) In silico test results

#### (i) Consumption test

The PWM parameters of the device after 10 min are shown in table 1, where $N_{\mathrm{falling}}$ is the number of falling edges, $N_{\mathrm{rising}}$ the number of rising edges, $f_{\min}$ the minimal frequency, $f_{\max}$ the maximal frequency, $f_{\mathrm{mean}}$ the average frequency, $N_{p(+)}$ the number of positive parts, $N_{p(-)}$ the number of negative parts and $D_{\mathrm{cycle}}$ the duty cycle.

**Table 1.** PWM parameters in a 10 min run

parameter	value
$N_{\mathrm{falling}}$	854
$N_{\mathrm{rising}}$	854
$f_{\min}$	30.3876 Hz
$f_{\max}$	30.4112 Hz
$f_{\mathrm{mean}}$	30.3998 Hz
$N_{p(+)}$	854
$N_{p(-)}$	854
$D_{\mathrm{cycle}}$	752.4778 m%

#### (ii) Stability testing

The correct working process of the device was tested prior to implantation as explained in §b.

The consumption readouts in the different test range from 4.4 μA when in sleep mode to 8.73 μA when working.

**Figure 6.**
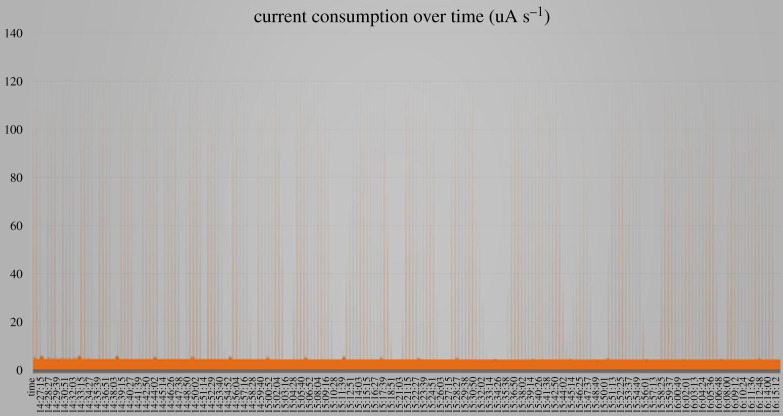
Current consumption during working and sleep cycles. (Online version in colour.)

In figure 6, a test run of 10 min is shown to evaluate the stability of the system.

### (f) *In vivo* test results

Following the procedure described above, we implanted our designed VNS system in 32 GASH/Sal for 14 days to carry out diverse experiments to study the mechanisms of action of VNS. We evaluated the presence and magnitude of the seizures before the surgery and then again 7, 10 and 14 days after the implantation of the PCB.

The animals were exposed to white noise (0–108 kHz and 115–120 dB) until the tonic-colonic seizure appeared (or during a minute maximum). The animal’s behaviour was recorded and used later for the neuroethological study by means of the Ethomatic software. The severity of the seizures was evaluated using the level Seizure Index.

At the end of the experiments, all the animals showed the total abolition of tonic-clonic seizures. Although a few of them retained wild running behaviour at the beginning of the stimulation, the Seizure Index was less than or equal to 3 (which is a very low index).

The neuroethological results obtained using our own VNS system are similar to those obtained with the use of a commercial device (VNS Therapy, Demipulse Duo, Model 104 Generator, Livanova) designed for clinical use. No adverse effects were observed in any of the animals studied.

## 4. Conclusion

In comparison with the average price of a VNS device, we have achieved a final cost of 150€ each device. This allows for a really affordable VNS system for animal experimentation and a great reduction of cost compared with the commercial versions available.

Also, the newly designed device represents a fraction of the size of a commercial VNS stimulator (table 2), making it easier to be surgically implanted and reducing the drawbacks of the surgery. To our knowledge, this is the first VNS device developed specifically for its use in rodents and animal experimentation. Moreover, the capsule made with BiomedClean resin (FormLabs) designed for the device allows for easy access to the electronics before the surgery and for repairs to be made in case these are deemed necessary.

**Table 2.** Size comparison between commercial VNS devices and the developed prototype; all measurements are in millimetres.

prototype	${\mathrm{SenTiva}\;\mathrm{Duo}}^{\mathrm{TM}}$ M1000-D	AspireSR${\;}^{®}$ M106	Demipulse${\;}^{®}$ Duo M104
20×20×3.2	45×39×6.9	52×52×6.9	45×39×6.3

The size differences can be attributed to the difference in battery life between the commercial VNS devices and the developed prototype. Since animal experimentation does not require the devices to run during the whole lifespan of the animal the battery life can be severely reduced from the usual 5–10 years in a commercial device to a year in the case of the prototype.

**Figure 7.**
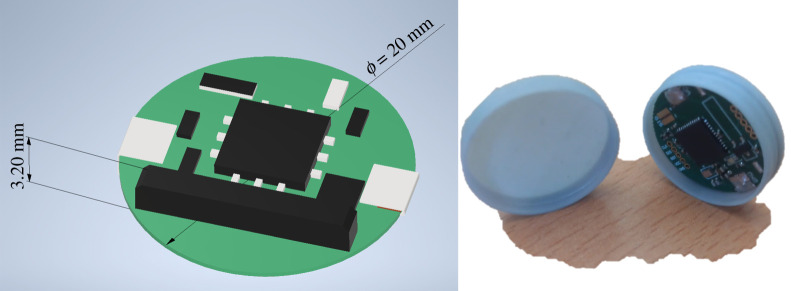
Rendering of the device with the button cell battery installed and photograph of the encapsulated device (without the cap installed). (Online version in colour.)

The final version of the device with the encapsulation (and the rendering of the electronics) can be seen in figure 7.

The design of the device has thus far exceeded the expectations that were originally set when the work began. The high efficacy in controlling epileptic seizures and the low price of the device have been a great success. However, there is still room for improvement in the device in two main areas: insulation and coating, and long-term performance.

Future developments should consider the implementation of certain features of commercial devices such as remote programming or modification of parameters once the device has been implanted. Furthermore, in the field of insulation, biocompatible materials should be sought that reduce the immunological response to foreign bodies.

## Data Availability

This article has no additional data.
